# Detection of Antibiotic Resistance, Virulence Gene, and Drug Resistance Gene of *Staphylococcus aureus* Isolates from Bovine Mastitis

**DOI:** 10.1128/spectrum.00471-22

**Published:** 2022-06-27

**Authors:** Zhe Zhang, Yun Chen, Xinpu Li, Xurong Wang, Hongsheng Li

**Affiliations:** a Lanzhou Institute of Husbandry and Pharmaceutical Sciences, Chinese Academy of Agricultural Sciences/KeyLab of Veterinary Pharmaceutical Development, Ministry of Agriculture and Rural Affairs/Engineering and Technology Research Center of Traditional Chinese Veterinary Medicine, Lanzhou, People’s Republic of China; INTHERES; China Agricultural University; Heilongjiang Bayi Agricultural University

**Keywords:** *Staphylococcus aureus*, antibiotic resistance, virulence gene, drug resistance gene

## Abstract

Antimicrobial therapy plays an important role in mastitis control caused by Staphylococcus aureus but has become less effective due to widespread drug resistance. The purpose of this study was to detect antibiotic resistance, drug resistance gene, and virulence gene of S. aureus strains. In this study, 2,962 milk samples were collected from 43 dairy farms located in 16 provinces of China and cultured for isolation of S. aureus. Antibiotic resistance, capsular polysaccharide, spa typing, virulence genes, and drug resistance genes of the strains were analyzed. Of 2,962 samples, 298 strains were isolated and identified as S. aureus. The strains exhibited high percentages of resistance to penicillin G (91.95%). Moreover, all strains showed resistance to more than one antimicrobial agent but were sensitive to nitrofurantoin and sulfamethoxazole/trimethoprim. The results indicate that type 8 was the dominant capsular polysaccharide serotype and t459 was the dominant spa type. The most prevalent virulence gene was *clfA* (98%). The resistance genes of several antibiotics were detected, among which the *blaZ* gene (92.95%) was the highest. In conclusion, we present the antimicrobial resistance and virulence genes of S. aureus in this study which are of importance for mastitis control.

**IMPORTANCE** Bovine mastitis is a serious disease associated with both high incidence and economic loss, posing a major challenge to the dairy industry worldwide. Staphylococcus aureus is one of the most common pathogens to cause bovine mastitis, and antimicrobial therapy plays an important role in mastitis control caused by S. aureus but has become less effective due to widespread drug resistance. The purpose of this study was to detect antibiotic resistance, drug resistance gene, and virulence gene of S. aureus strains, which would be helpful to mastitis control.

## INTRODUCTION

Bovine mastitis is a serious disease associated with both high incidence and economic loss, posing a major challenge to the dairy industry worldwide (1, 2). The global economic loss due to mastitis was estimated to be $35 billion per year, including reduced milk production, condemnation of milk due to antibiotic residues, veterinary costs, culling of chronically infected cows, and occasional deaths (3). Moreover, mastitis poses a threat to human health since it may be responsible for zoonoses and for food toxin infections (4).

Mastitis is complex, developing as a result of the interaction between various factors associated with the host, specific pathogens, environment, and management (5). Over 200 different organisms have been recorded to cause bovine mastitis (6). Among the pathogenic bacteria, Staphylococcus aureus is one of the most common pathogens to cause bovine mastitis, especially in China (7–9). Antibiotic treatment is a key component for treatment of diseases caused by S. aureus. However, the abuse of antibiotics has led to the resistance of bacteria to drugs in recent years, and the issue of multidrug resistance has become increasingly prominent (10). There is a close relationship between bacterial resistance and drug resistance genes. *mecA* gene is considered one of the major resistance genes that confer resistance to β-lactams (11). In addition, *blaZ* gene also plays an important role in beta-lactam resistance in S. aureus (12).

At the same time, due to the toxicity and infectivity of S. aureus, the harm of S. aureus is stronger. This is related to the virulence genes the bacteria contain; S. aureus attaches to epithelial cells of the teat canal depending on the interaction of bacterial surface proteins, such as clumping factors A and B (*clfA* and *clfB*) and fibronectin-binding protein A (*fnbA*), providing assurance that the bacteria further infect the mammary gland (13, 14). Enterotoxin is an important virulence means of S. aureus; more than 20 different enterotoxins have been characterized, and these enterotoxins have been traditionally subdivided into classical genes (*sea* to *see*), which have been well distinguished and used in various detection methods. In addition, the toxic shock syndrome toxin TSST-1 and staphylococcal exfoliative toxin (ET) are important causes of mastitis with S. aureus (15, 16).

The aim of the present study was to investigate the relevant situation of S. aureus in dairy cow mastitis and to characterize these strains by the corresponding antibiotic drug sensitivity test, through evaluation of the genes of the drug resistance and the virulence by PCR molecular analyses.

## RESULTS

### Isolation and identification of S. aureus strains.

A total of 298 S. aureus strains were isolated from 2,962 mastitis samples as shown in Table 1.

**TABLE 1:** Distribution of S. aureus isolates

Province	No. of samples	No. of strains	Isolation rate
Gansu	451	53	11.75%
Jiangsu	237	11	4.64%
Shandong	227	20	8.81%
Shanxi	223	26	11.66%
Xinjiang	216	25	11.57%
Heilongjiang	195	18	9.23%
Ningxia	182	15	8.24%
Inner Mongolia	166	22	13.25%
Sichuan	166	17	10.24%
Guizhou	151	18	11.92%
Jilin	147	11	7.48%
Hebei	136	14	10.29%
Qinghai	134	20	14.93%
Henan	121	13	10.74%
Shaanxi	112	6	5.36%
Hubei	98	9	9.18%
In total	2962	298	10.06%

### Antimicrobial susceptibility testing.

As shown in Table 2, the 298 S. aureus strains were tested for resistance to 11 antimicrobial agents by using the K-B disk diffusion method. Antimicrobial resistance was observed most frequently to penicillin G (91.95%), and all bacteria are resistant to at least two antibiotics (100%). However, all strains were sensitive to nitrofurantoin and sulfamethoxazole/trimethoprim.

**TABLE 2:** Results of 298 strains of S. aureus in drug resistance experiments

Antibiotic class	Antibiotic	Strains, no. (%)
β-Lactam	Penicillin G	274 (91.95)
	Cefoxitin	77 (25.84)
Aminoglycoside	Gentamicin	159 (53.86)
	Kanamycin	194 (65.10)
Lincosamide	Clindamycin	167 (56.04)
Quinolone	Ciprofloxacin	146 (48.99)
	Levofloxacin	146 (48.99)
Chloramphenicol	Chloramphenicol	26 (8.72)
Nitrofuran	Nitrofurantoin	0 (0)
Chain-positive bacteriocin	Quinupristin/dalfopristin	3 (1.01)
Rifamycin	Rifampicin	54 (18.12)
Tetracycline	Tetracycline	73 (24.50)
Sulfonamide	Sulfamethoxazole/trimethoprim	0 (0)
Oxazolidinone	Linezolid	1 (0.34)
Multidrug resistant		298 (100)

### Bacterial typing.

The results of capsular polysaccharide typing test indicated that cp8 (57.38%) S. aureus was the most popular strain in China, followed by cp5 (35.57%) and cp336 (7.05%) as shown in Table 3.

**TABLE 3:** Capsular polysaccharide typing of S. aureus

Bacterial typing	Strains, no. (%)
cp5	106 (35.57)
cp8	171 (57.38)
cp336	21 (7.05)

A total of 48 spa types were identified within 298 strains. The most prevalent spa type was t459 (18.79%), followed by t6367 (16.78%), t067 (9.40%), t163 (6.38%), t4904 (4.03%), t13751 (3.69%), et al. (Table 4).

**TABLE 4:** Spa typing of S. aureus

Bacterial typing	Strains, no. (%)
t459	56 (18.79)
t6367	50 (16.78)
t458	3 (1.00)
t7073	9 (3.02)
t3932	10 (3.36)
t18401	3 (1.00)
t163	19 (6.38)
t304	3 (1.00)
t6811	5 (1.68)
t4904	12 (4.03)
t067	28 (9.40)
t9531	3 (1.00)
t6379	2 (0.67)
t2524	3 (1.00)
t3867	1 (0.34)
t4652	3 (1.00)
t7880	7 (2.35)
t3592	6 (2.01)
t17343	2 (0.67)
t521	6 (2.01)
t195	5 (1.68)
t6272	2 (0.67)
t3626	5 (1.68)
t527	7 (2.35)
t13751	11 (3.69)
t2246	1 (0.34)
t14061	1 (0.34)
t808	4 (1.34)
t1456	3 (1.00)
t1250	7 (2.35)
t4558	3 (1.00)
t9537	1 (0.34)
t14605	1 (0.34)
t1987	2 (0.67)
t12238	1 (0.34)
t870	1 (0.34)
t342	1 (0.34)
t10555	1 (0.34)
t1521	1 (0.34)
t14936	1 (0.34)
t5355	1 (0.34)
t6159	1 (0.34)
t079	1 (0.34)
t026	1 (0.34)
t233	1 (0.34)
t421	1 (0.34)
t3111	1 (0.34)
t4976	1 (0.34)

### Antimicrobial resistance genes.

In the experiments of antimicrobial resistance genes, the highest frequency gene we isolated was *blaZ* gene (92.95%), followed by *aacA-aphD* (87.25%), *tetK* (48.66%), *tetM* (27.85%), *norA* (55.03%), *norB* (53.36%), and *norC* (57.71%). As a key gene for methicillin-resistant S. aureus, 73 strains of *mecA* gene were detected and confirmed by PBP2a gel experiment (Table 5).

**TABLE 5:** Status of antibiotic resistance-related genes in S. aureus

Antibiotic	Gene	Strains, no. (%)
Tetracycline	*tetK*	145 (48.66)
	*tetM*	83 (27.85)
Aminoglycosides	*aacA-aphD*	260 (87.25)
β-Lactams	*blaZ*	277 (92.95)
	*mecA*	73 (24.50)
	*norA*	164 (55.03)
Quinolones	*norB*	159 (53.36)
	*norC*	172 (57.71)

### Detection of virulence determinants.

Among 298 strains of S. aureus, we detected genes such as adhesion factors, enterotoxin, toxic shock syndrome toxin, and exfoliative toxins, among which the *clfA* (97.99%) has the highest frequency, leaving the *clfB* (96.64%), *fnbA* (96.64%), *ebpS* (36.56%), *sea* (17.45%), *seb* (16.44%), *sec* (7.38%), *sed* (1.68%), *see* (0%), *tst* (23.50%), *eta* (1.34%), and *etb* (0%) (Table 6).

**TABLE 6:** Detection of virulence determinants of S. aureus

Toxin category	Gene	Strains, no. (%)
Adhesion factor	*fnbA*	288 (96.64)
	*clfA*	292 (97.99)
	*clfB*	288 (96.64)
	*ebpS*	109 (36.56)
Enterotoxin	*sea*	52 (17.45)
	*seb*	49 (16.44)
	*sec*	22 (7.38)
	*sed*	5 (1.68)
	*see*	0 (0)
Toxic shock syndrome toxin	*tst*	70 (23.50)
Exfoliative toxin	*eta*	4 (1.34)
	*etb*	0 (0)

## DISCUSSION

S. aureus represents a major agent of contagious bovine mastitis. Our study indicated that 10.06% (298/2962) of mastitis samples were positive for S. aureus, which is significantly lower than previous reports by Liu et al. (17) and Zhang et al. (18) (27.7% and 29%). However, our data are similar to those of the report by Seo et al. (11.6%, 40/345) (19). In addition, 2% to 50% and even higher prevalence of S. aureus mammary gland infection was also observed in another report (20). Overall, S. aureus is still very common in China.

The resistance of S. aureus to antimicrobial agents is an increasing global problem. A drug sensitivity test is required not only for effective therapy but also for monitoring the spread of resistant strains. Eleven antimicrobial agents were used in this study, and all isolates were resistant to at least two antibiotics, especially to penicillin G (91.95%). Penicillin is a well-known antibiotic that is widely used in clinical practice, leading to the general resistance of S. aureus. We found that 91.95% of S. aureus isolates were resistant to penicillin, which was similar to earlier reports from China by Liu et al. (17), who reported that 85.2% of S. aureus isolates exhibited resistance to penicillin G, and Jian-Ning et al. (21), who reported 94.6%. However, Haran et al. (22) proved that 16% of 93 S. aureus isolates from 42 farms in America were resistant to penicillin. Another report from New Zealand in 2014 (23) found that of 364 S. aureus isolates, 28% were resistant to penicillin. Our data indicated that penicillin-resistant S. aureus in Chinese dairy farms is more common than that in other countries.

At the same time, we found that the resistance rates of kanamycin (65.10%), clindamycin (56.04%), gentamicin (53.86%), ciprofloxacin (48.99%), and levofloxacin (48.99%) were also very high. In contrast, these isolates had low drug resistance to quinupristin/dalfopristin (1.01%), linezolid (0.34%), nitrofurantoin (0%), and sulfamethoxazole/trimethoprim (0%). The clinical use of penicillin, kanamycin, clindamycin, ciprofloxacin, and levofloxacin should be minimized. As nitrofurantoin was prohibited for use in food-producing animals in China, sulfamethoxazole/trimethoprim should be considered preferentially for the treatment of bovine mastitis caused by S. aureus. It is recommended to perform a drug sensitivity test before using an antibiotic drug (21).

The presence of resistance-associated genes in S. aureus was detected in this study. Resistance to penicillin is caused mainly by the *blaZ* gene encoding production of beta-lactamases, which hydrolytically destroy beta-lactams (24). An antimicrobial resistance genes test revealed that 92.95% of isolates were found carrying *blaZ* genes, agreeing with the finding that 91.95% of isolates were resistant to penicillin G. This is in agreement with data from Olsen et al. (25), showing that all penicillin-resistant strains carried *blaZ*. The activated *blaZ* could encode β-lactamase enzyme (penicillinase), which inactivates the antibiotic through hydrolysis of the peptide bond in the β-lactam ring (26). Methicillin-resistant Staphylococcus aureus (MRSA) is a growing concern worldwide and has increasingly been recognized in farm animal populations in recent years (11, 22, 27). MRSA isolates are frequently multidrug resistant (MDR), which can result in higher costs, longer treatment times, and higher rates of hospitalization and comorbidities. Presence of *mecA* gene is generally recognized as the most reliable method for detection of methicillin resistance. In this study, 73 strains (24.50%) positive for the *mecA* gene were detected and confirmed by PBP2a gel experiment. However, some previous reports from China revealed lower isolation rates of MRSA with 15.53% (34/219) (28) and 14.20% (22/155) (29), which indicated the increasing presence of MRSA isolates in Chinese dairy cattle. Therefore, careful monitoring of the resistance status of S. aureus in dairy environments is needed, as the presence of MRSA poses potential risk to farm workers, veterinarians, and farm animals (17, 22).

Capsular polysaccharide serotyping of S. aureus was first reported in 1982 (30), and 11 serotypes have been described. Our results showed that cp8 (57.38%) S. aureus was the most popular strain in Chinese dairy farms, followed by cp5 (35.57%) and cp336 (7.05%). The existence of capsular polysaccharides in S. aureus plays an important role in the pathogenicity and immunogenicity. Our data provide a reference for the research and development of bovine mastitis vaccine. Molecular characterization of S. aureus is vital for the rapid identification of prevalent strains and will contribute to the control and prevention of S. aureus. Spa typing, which relies only on the assessment of the number of and sequence variation in repeats at the x region of the spa gene, exhibits excellent discriminatory power and has become a useful typing tool for the sake of its ease of performance, less expensive procedure, and standardized nomenclature (31). In our study, a total of 48 spa types were identified within 298 strains, of which t459 (18.79%) was the most prevalent spa type, followed by t6367 (16.78%), t067 (9.40%), t163 (6.38%), t4904 (4.03%), t13751 (3.69%), et al. A previous study reported that spa t224 (30.4%) was the most common type in Ningxia province of China (32). However, another study demonstrated that t267 (35.84%) was the predominant spa type in Liaoning province of China (33). Interestingly, t224 and t267 spa types were not detected at all in our study. Our results therefore suggest that the distribution of spa types varies between the different regions of China.

The broad range of infections caused by S. aureus is related to a number of virulence factors that allow it to adhere to surfaces, invade or avoid the immune system, and cause harmful toxic effects to the host (34). Adhesins are considered the most important virulence factors during early phases of S. aureus infection. The fibronectin-binding proteins (FnbP) and clumping factor proteins (*clfA*, *clfB*) can promote adhesion of S. aureus cells to a variety of molecules and surfaces, and they have been implicated in cell-cell adhesion. The *fnbA* and *clfB* genes were detected in almost all 298 isolates, which approaches the data reported by Wei et al. (35). Enterotoxin is a class of low-molecular-weight proteins with superantigen activity and is highly resistant to denaturation. In our research, *sea* gene (17.45%) was the most frequent, and the rest were *seb* (16.44%), *sec* (7.38%), and *sed* (1.68%). The *see* gene was not detected in any of the isolates. Some studies report the prevalence of the *sea* and *sed* genes in 10% and 7.50% of isolates (36), and another reports 53.30% prevalence for *sea*, 3.30% for *seb*, 50% for *sec*, 4% for *sed*, and 46.60% for *see* in isolates of S. aureus of mastitis milk (37). This indicates that the enterotoxin gene content of bacteria is different in different regions. Toxic shock syndrome toxin TSST-1 and staphylococcal toxin (ETs) are also an important part of S. aureus virulence. *tst* (23.50%) was also detected in a lower percentage than other genes in this study and was not found in the study reported in reference 38.

**Conclusions.** Our study indicated that 10.06% (298/2962) of mastitis samples from China were positive for S. aureus, and all isolates (100%) were resistant to at least two antibiotics, especially to penicillin G (91.95%). Sulfamethoxazole/trimethoprim should be considered preferentially for the treatment of bovine mastitis caused by S. aureus. Type 8 (57.38%) was the dominant capsular polysaccharide serotype, and t459 (18.79%) was the dominant spa type. The resistance genes of several antibiotics were detected, of which *blaZ* gene (92.95%) was highest, and the most prevalent virulence gene was *clfA* (98%).

## MATERIALS AND METHODS

### Collection of samples.

From 2016 to 2020, a total of 2,962 mastitis samples were collected from 43 large-scale dairy farms located in 16 provinces of China as shown in Table 1. Most of them (*n* = 2,370) were collected from clinical mastitis cows, and 592 mastitis samples were collected from subclinical mastitis cows. After disinfecting with 75% alcohol, collected milk samples were stored in cold storage and sent to the laboratory for bacterial isolation and identification.

### Isolation and identification.

Samples were inoculated in blood agar plates (Huankai Microbial Sci&Tech co., Ltd., Guangdong, China), and a typical single colony from each sample was picked for purification. Purified bacteria were subjected to smearing, Gram staining, and microscopic examination using the CHROM agar chromogenic medium for isolation and direct differentiation of S. aureus (Shanghai Central Bio-engineering Co., Ltd., Shanghai, China). Genetic testing was performed using a TakaLa 16S rRNA gene bacterial identification PCR kit (TaKaRa Biomedical Technology Co., Ltd., Dalian, China). Purified products were sent to the sequencing company, and the sequencing results were analyzed and determined by the BLAST program on the NCBI website.

### Antimicrobial resistance.

The drug sensitivity test was carried out according to the standards of the American Committee for Clinical Laboratory Standards using the K-B disk diffusion method (39). Penicillin G (10 μg), cefoxitin (30 μg), ciprofloxacin (5 μg), levofloxacin (5 μg), gentamicin (10 μg), kanamycin (30 μg), clindamycin (2 μg), chloramphenicol (30 μg), nitrofurantoin (30 μg), quinupristin/dalfopristin (15 μg), rifampin (5 μg), tetracycline (30 μg), sulfamethoxazole/trimethoprim (25 μg), and linezolid (30 μg) were used as antimicrobial agents (Oxoid, Basingstoke, UK).

### Bacterial typing.

Both capsular polysaccharide typing and spa typing were carried out on all strains. According to Verdier et al. (40), Cap5 k1 (5′-GTCAAAGATTATGTGATGCTACTGAG-3′), Cap5 k2 (5′-ACTTCGAATATAAACTTGAATCAATGTTATACAG-3′) located in cap5k for capsular type 5 and cap8 k1 (5′-GCCTTATGTTAGGTGATAAACC-3′), cap8 k2 (5′-GGAAAAACACTATCATAGCAGG-3′) located in cap8I were synthesized. The polymorphic X region of the protein A gene (spa) was amplified using the primers spa-1113f (5′ TAA AGA CGA TCC TTC GGT GAG C 3′) and spa-1514r (5′ CAG CAG TAG TGC CGT TTG CTT 3′). The results were submitted to the website for further detection (https://www.spaserver.ridom.de) (41).

### Antimicrobial resistance genes.

According to the results of the drug sensitivity test, the related genes of several antibiotics with the highest drug resistance were selected. Penicillin (*mecA*, *blaZ*) (42), aminoglycosides (*aac*) (43), tetracycline (*tetK*, *tetM*) (14, 42), and quinolones (*norA*, *norB*, *norC*) (44) were detected by PCR. For *mecA*-positive strains, methicillin-resistant S. aureus (MRSA) was confirmed by PBP2a gel test.

### Detection of virulence determinants.

The genes encoding staphylococcal adhesion factor (*fnbA*, *clfA*, *clfB*, *ebpS*), enterotoxin (*sea*, *seb*, *sec*, *sed*, *see*), poisoning syndrome toxin (*tst*), and shedding toxin (*eta*, *etb*) were tested by PCR in this study (45, 46).

## Supplementary Material

Reviewer comments

## References

[1] Zhang Q, Xing S, Sun Q, Pei G, Cheng S, Liu Y, An X, Zhang X, Qu Y, Tong Y. 2017. Characterization and complete genome sequence analysis of a novel virulent Siphoviridae phage against *Staphylococcus aureus* isolated from bovine mastitis in Xinjiang, China. Virus Genes 53:464–476. doi:10.1007/s11262-017-1445-z. 28299517

[2] Enger B, Nickerson S, Tucker H, Parsons C, Akers R. 2019. Apoptosis and proliferation in *Staphylococcus aureus*-challenged, nonlactating mammary glands stimulated to grow rapidly and develop with estradiol and progesterone. J Dairy Sci 102:857–865. doi:10.3168/jds.2018-15498. 30415855

[3] Abebe R, Hatiya H, Abera M, Megersa B, Asmare K. 2016. Bovine mastitis: prevalence, risk factors and isolation of*Staphylococcus aureus* in dairy herds at Hawassa milk shed, South Ethiopia. BMC Vet Res 12:270. doi:10.1186/s12917-016-0905-3. 27912754 PMC5135792

[4] Bhandari S, Subedi D, Tiwari BB, Shrestha P, Shah S, Al-Mustapha AI. 2021. Prevalence and risk factors for multidrug-resistant Escherichia coli isolated from subclinical mastitis in the western Chitwan region of Nepal - ScienceDirect. J Dairy Sci 104:12765–12772. doi:10.3168/jds.2020-19480. 33838890

[5] Harjanti DW, Ciptaningtyas R, Wahyono F, Setiatin E. 2018. Isolation and identification of bacterial pathogen from mastitis milk in Central Java Indonesia. IOP Conf Ser: Earth Environ Sci 102:e012076. doi:10.1088/1755-1315/102/1/012076.

[6] Blowey R, Edmondson P. 2010. Mastitis control in dairy herds, 18–20. CABI, Wallingford, UK.

[7] Zhang Z, Li XP, Yang F, Luo JY, Wang XR, Liu LH, Li HS. 2016. Influences of season, parity, lactation, udder area, milk yield, and clinical symptoms on intramammary infection in dairy cows. J Dairy Sci 99:6484–6493. doi:10.3168/jds.2016-10932. 27265170

[8] Attili A, Nebbia P, Bellato A, Galosi L, Papeschi C, Rossi G, Linardi M, Fileni E, Cuteri V, Chiesa F, Robino P. 2020. The effect of age and sampling site on the outcome of *Staphylococcus aureus* infection in a rabbit (Oryctolagus cuniculus) farm in Italy. Animals 10:774. doi:10.3390/ani10050774. PMC7278480 32365654

[9] Demontier E, Dubé-Duquette A, Brouillette E, Larose A, Ster C, Lucier J-F, Rodrigue S, Park S, Jung D, Ruffini J, Ronholm J, Dufour S, Roy J-P, Ramanathan S, Malouin F. 2021. Relative virulence of *Staphylococcus aureus* bovine mastitis strains representing the main Canadian spa types and clonal complexes as determined using in vitro and in vivo mastitis models. J Dairy Sci 104:11904–11921. doi:10.3168/jds.2020-19904. 34454755

[10] Rabello RF, Bonelli RR, Penna BA, Albuquerque JP, Souza RM, Cerqueira AMF. 2020. Antimicrobial resistance in farm animals in Brazil: an update overview. Animals 10:552. doi:10.3390/ani10040552. PMC7222418 32224900

[11] Klibi A, Jouini A, Gómez P, Slimene K, Ceballos S, Torres C, Maaroufi A. 2018. Molecular characterization and clonal diversity of methicillin-resistant and -susceptible *Staphylococcus aureus* isolates of milk of cows with clinical mastitis in Tunisia. Microb Drug Resist 24:1210–1216. doi:10.1089/mdr.2017.0278. 29373088

[12] Martini CL, Lange CC, Brito MA, Ribeiro JB, Mendonça LC, Vaz EK. 2017. Characterisation of penicillin and tetracycline resistance in *Staphylococcus aureus* isolated from bovine milk samples in Minas Gerais, Brazil. J Dairy Res 84:202–205. doi:10.1017/S0022029917000061. 28290267

[13] Stutz K, Stephan R, Tasara T. 2011. SpA, ClfA, and FnbA genetic variations lead to Staphaurex test-negative phenotypes in bovine mastitis *Staphylococcus aureus* isolates. J Clin Microbiol 49:638–646. doi:10.1128/JCM.01148-10. 21147952 PMC3043514

[14] Bochniarz M, Adaszek Ł, Dzięgiel B, Nowaczek A, Wawron W, Dąbrowski R, Szczubiał M, Winiarczyk S. 2016. Factors responsible for subclinical mastitis in cows caused by Staphylococcus chromogenes and its susceptibility to antibiotics based on bap, fnbA, eno, mecA, tetK, and ermA genes. J Dairy Sci 99:9514–9520. doi:10.3168/jds.2016-11723. 27692714

[15] Costa FN, Belo NO, Costa EA, Andrade GI, Pereira LS, Carvalho IA, Santos RL. 2018. Frequency of enterotoxins, toxic shock syndrome toxin-1, and biofilm formation genes in *Staphylococcus aureus* isolates from cows with mastitis in the Northeast of Brazil. Trop Anim Health Prod 50:1089–1097. doi:10.1007/s11250-018-1534-6. 29429115

[16] Mohseni M, Rafiei F, Ghaemi EA. 2018. High frequency of exfoliative toxin genes among *Staphylococcus aureus* isolated from clinical specimens in the north of Iran: alarm for the health of individuals under risk. Iran J Microbiol 10:158–165. 30112153 PMC6087700

[17] Liu H, Li S, Meng L, Dong L, Zhao S, Lan X, Wang J, Zheng N. 2017. Prevalence, antimicrobial susceptibility, and molecular characterization of *Staphylococcus aureus* isolated from dairy herds in northern China. J Dairy Sci 100:8796–8803. doi:10.3168/jds.2017-13370. 28865851

[18] Zhang L, Li Y, Bao H, Wei R, Zhou Y, Zhang H, Wang R. 2016. Population structure and antimicrobial profile of *Staphylococcus aureus* strains associated with bovine mastitis in China. Microb Pathog 97:103–109. doi:10.1016/j.micpath.2016.06.005. 27265679

[19] Seo YH, Jang JH, Moon KD. 2010. Occurrence and characterization of enterotoxigenic *Staphylococcus aureus* isolated from minimally processed vegetables and sprouts in Korea. Food Sci Biotechnol 19:313–319. doi:10.1007/s10068-010-0045-7.

[20] Miroslav B, Habrun B, Kompes G. 2012. Clinical and epidemiological aspects of cow mastitis caused by *S. aureus* and its methicillin-resistant strains. Rad Hazu Med Znan 37:113–118.

[21] Jian-Ning WU, Jia-Yin WU, Lin J. 2011. Antibiotics resistance in 74 strains of *Staphylococcus aureus* from acute mastitis. Chin J Micro 8:38–42.

[22] Haran KP, Godden SM, Boxrud D, Jawahir S, Bender JB, Sreevatsan S. 2012. Prevalence and characterization of *Staphylococcus aureus*, including methicillin-resistant *Staphylococcus aureus*, isolated from bulk tank milk from Minnesota dairy farms. J Clin Microbiol 50:688–695. doi:10.1128/JCM.05214-11. 22170937 PMC3295154

[23] Mcdougall S, Hussein H, Petrovski KR. 2014. Antimicrobial resistance in *Staphylococcus aureus*, Streptococcus uberis and Streptococcus dysgalactiae from dairy cows with mastitis. N Z Vet J 62:68–76. doi:10.1080/00480169.2013.843135. 24215609

[24] Pinho MG. 2008. Mechanisms of beta-lactam and glycopeptide resistance in *Staphylococcus aureus*. Staph Mol Genet 207–226.

[25] Olsen JE, Christensen H, Aarestrup FM. 2006. Diversity and evolution of blaZ from *Staphylococcus aureus* and coagulase-negative staphylococci. J Antimicrob Chemother 57:450–460. doi:10.1093/jac/dki492. 16449305

[26] Jensen SO, Lyon BR. 2009. Genetics of antimicrobial resistance in *Staphylococcus aureus*. Future Microbiol 4:565–582. doi:10.2217/fmb.09.30. 19492967

[27] Vestergaard M, Frees D, Ingmer H. 2019. Antibiotic resistance and the MRSA problem. Microbiol Spectr 7:1–23. doi:10.1128/microbiolspec.GPP3-0057-2018. PMC11590431 30900543

[28] Wang D, Wang Z, Yan Z, Wu J, Ali T, Li J, Lv Y, Han B. 2015. Bovine mastitis *Staphylococcus aureus*: antibiotic susceptibility profile, resistance genes and molecular typing of methicillin-resistant and methicillin-sensitive strains in China. Infect Genet Evol 31:9–16. doi:10.1016/j.meegid.2014.12.039. 25582604

[29] Dan M, Yehui W, Qingling M, Jun Q, Xingxing Z, Shuai M, Kuojun C, Jinsheng Z, Zibing C, Zaichao Z, Xuepeng C. 2019. Antimicrobial resistance, virulence gene profile and molecular typing of *Staphylococcus aureus* isolates from dairy cows in Xinjiang Province, northwest China. J Glob Antimicrob Resist 16:98–104. doi:10.1016/j.jgar.2018.08.024. 30213718

[30] Karakawa WW, Vann WF. 1982. Capsular polysaccharides of *Staphylococcus aureus*. Semin Infect Dis 4:285–293.

[31] Asadollahi P, Farahani NN, Mirzaii M, Khoramrooz SS, Van Belkum A, Asadollahi K, Dadashi M, Darban-Sarokhalil D. 2018. Distribution of the most prevalent spa types among clinical isolates of methicillin-resistant and susceptible *Staphylococcus aureus* around the world: a review. Front Microbiol 9:163–179. doi:10.3389/fmicb.2018.00163. 29487578 PMC5816571

[32] Chen C, Sun C, Li J, Ji X, Wang Y, Song C, Wang G. 2021. Characterisation of *Staphylococcus aureus* isolates from bovine mastitis in Ningxia, Western China. J Glob Antimicrob Resist 25:232–237. doi:10.1016/j.jgar.2021.03.021. 33866044

[33] Zhang D, Li Y, Yang X, Su HY, Wang Q, Zhang ZH, Liu YC, Tian CL, Cui CC, Liu MC. 2020. In vitro antibiotic susceptibility, virulence genes distribution and biofilm production of *Staphylococcus aureus* isolates from bovine mastitis in the Liaoning Province of China. Infect Drug Resist 13:1365–1375. doi:10.2147/IDR.S247765. 32494168 PMC7234830

[34] Bien J, Sokolova O, Bozko P, Williams HD. 2011. Characterization of virulence factors of *Staphylococcus aureus*: novel function of known virulence factors that are implicated in activation of airway epithelial proinflammatory response. J Pathog 2011:601905. doi:10.4061/2011/601905. 22567334 PMC3335658

[35] Wei W, Xiaohui L, Tao J, Zixin P, Jin X, Lingxian Y, Fengqin L, Séamus F, Zulqarnain B. 2018. Prevalence and characterization of *Staphylococcus aureus* cultured from raw milk taken from dairy cows with mastitis in Beijing, China. Front Microbiol 9:1123. doi:10.3389/fmicb.2018.01123. 29988423 PMC6024008

[36] Khoramrooz SS, Mansouri F, Marashifard M, Malek Hosseini SAA, Akbarian Chenarestane-Olia F, Ganavehei B, Gharibpour F, Shahbazi A, Mirzaii M, Darban-Sarokhalil D. 2016. Detection of biofilm related genes, classical enterotoxin genes and agr typing among *Staphylococcus aureus* isolated from bovine with subclinical mastitis in southwest of Iran. Microb Pathog 97:45–51. doi:10.1016/j.micpath.2016.05.022. 27251096

[37] Fursova K, Shchannikova M, Loskutova I, Shepelyakovskaya A, Laman A, Boutanaev A, Sokolov S, Artem'eva O, Nikanova D, Zinovieva N, Brovko F. 2018. Exotoxin diversity of *Staphylococcus aureus* isolated from milk of cows with subclinical mastitis in Central Russia. J Dairy Sci 101:4325–4331. doi:10.3168/jds.2017-14074. 29477514

[38] Mello PM, Riboli DFM, Pinheiro L, Martins LDA, Paiva Brito MPV, da Cunha MDLRDS. 2016. Detection of enterotoxigenic potential and determination of clonal profile in *Staphylococcus aureus* and coagulase-negative staphylococci isolated from bovine subclinical mastitis in different Brazilian states. Toxins (Basel) 8:104. doi:10.3390/toxins8040104. 27092525 PMC4848630

[39] Chu C, Wei Y, Chuang S-T, Yu C, Changchien C-H, Su Y. 2013. Differences in virulence genes and genome patterns of mastitis-associated *Staphylococcus aureus* among goat, cow, and human isolates in Taiwan. Foodborne Pathog Dis 10:256–262. doi:10.1089/fpd.2012.1278. 23489048

[40] Verdier I, Durand G, Bes M, Taylor KL, Lina G, Vandenesch F, Fattom AI, Etienne J. 2007. Identification of the capsular polysaccharides in *Staphylococcus aureus* clinical isolates by PCR and agglutination tests. J Clin Microbiol 45:725–729. doi:10.1128/JCM.01572-06. 17202275 PMC1829147

[41] Strommenger B, Kettlitz C, Weniger T, Harmsen D, Friedrich AW, Witte W. 2006. Assignment of staphylococcus isolates to groups by spa typing, SmaI macrorestriction analysis, and multilocus sequence typing. J Clin Microbiol 44:2533–2540. doi:10.1128/JCM.00420-06. 16825376 PMC1489514

[42] Yang F, Wang Q, Wang XR, Wang L, Li XP, Luo JY, Zhang SD, Li HS. 2016. Genetic characterization of antimicrobial resistance in *Staphylococcus aureus* isolated from bovine mastitis cases in Northwest China. J Integr Agric 15:2842–2847. doi:10.1016/S2095-3119(16)61368-0.

[43] Strommenger B, Kettlitz C, Werner G, Witte W. 2003. Multiplex PCR assay for simultaneous detection of nine clinically relevant antibiotic resistance genes in *Staphylococcus aureus*. J Clin Microbiol 41:4089–4094. doi:10.1128/JCM.41.9.4089-4094.2003. 12958230 PMC193808

[44] Haubert L, Kroning IS, Iglesias MA, Padilha da Silva W. 2017. First report of the *Staphylococcus aureus* isolate from subclinical bovine mastitis in the South of Brazil harboring resistance gene dfrG and transposon family Tn 916–1545. Microb Pathog 113:242–247. doi:10.1016/j.micpath.2017.10.022. 29051059

[45] Peacock SJ, Moore CE, Justice A, Kantzanou M, Story L, Mackie K, O'neill G, Day NPJ. 2002. Virulent combinations of adhesin and toxin genes in natural populations of *Staphylococcus aureus*. Infect Immun 70:4987–4996. doi:10.1128/IAI.70.9.4987-4996.2002. 12183545 PMC128268

[46] Jarraud S, Mougel C, Thioulouse J, Lina G, Meugnier H, Forey F, Nesme X, Etienne J, Vandenesch F. 2002. Relationships between *Staphylococcus aureus* genetic background, virulence factors, agr groups (alleles), and human disease. Infect Immun 70:631–641. doi:10.1128/IAI.70.2.631-641.2002. 11796592 PMC127674

